# *SMAD7* loci contribute to risk of hepatocellular carcinoma and clinicopathologic development among Chinese Han population

**DOI:** 10.18632/oncotarget.8065

**Published:** 2016-03-14

**Authors:** Jiansong Ji, Min Xu, Zhongwei Zhao, Jianfei Tu, Jun Gao, Chenying Lu, Jingjing Song, Weiqian Chen, Minjiang Chen, Xiaoxi Fan, Xingyao Cheng, Xilin Lan, Jie Li

**Affiliations:** ^1^ Department of Radiology, The Fifth Affiliated Hospital of Wenzhou Medical University/Affiliated Lishui Hospital of Zhejiang University/The Central Hospital of Zhejiang Lishui, Lishui, Zhejiang, P.R. China; ^2^ Department of Hepatobiliary Surgery, Beijing Chao-yang Hospital Affiliated with Capital Medical University, Beijing, P.R. China; ^3^ The First Affiliated Hospital, Chongqing Medical University, Chongqing, P.R. China

**Keywords:** HCC, mothers against decapentaplegic homolog 7 (SMAD7), transforming growth factor beta (TGF-β), risk, SNP

## Abstract

Genome-wide association studies (GWAS) have identified three loci at 18q21 (rs4939827, rs7240004, and rs7229639), which maps to *SMAD7* loci, were associated with risk of diseases of the digestive system. However, their associations with hepatocellular carcinoma (HCC) risk remain unknown. A case-control study was conducted to assess genetic associations with HCC risk and clinicopathologic development among Chinese Han population. Three SNPs were genotyped among 1,000 HCC cases and 1,000 controls using Sequenom Mass-ARRAY technology. We observed statistically significant associations for the three *SMAD7* loci and HCC risk. Each copy of minor allele was associated with a 1.24–1.36 fold increased risk of HCC. We also found that significant differences were observed between rs4939827 and clinical TNM stage and vascular invasion, as well as rs7240004 and vascular invasion. We also established a genetic risk score (GRS) by summing the risk alleles. The GRS was significantly associated with increased risk of HCC and vascular invasion. Our data revealed the *SMAD7* loci is associated with HCC susceptibility and its clinicopathologic development.

## INTRODUCTION

Recent progress through the application of genome-wide association studies (GWAS) have identified a number of common variants involved in the etiology of hepatocellular carcinoma (HCC) [1, 2]. While various genome-wide significant findings have been reported previously, it remains likely that a substantial number of additional SNPs that did not satisfy the highly stringent (Bonferroni) statistical threshold may nonetheless be important factors in modifying disease risk, if for example, their main effects were operative only in certain sub-groups of the overall population. Understanding the effects of these variants in different populations is extremely important in terms of inferring the causality and mechanisms of HCC tumorigenesis, as well as for the translation of these results into risk prediction in different populations.

HCC is a disease with very different incidence rates between populations [3–5]. The risk variants may confer different magnitudes of increased risk in different populations for a variety of reasons, including differences in allele frequency and linkage disequilibrium (LD) structure, and differences in genetic and environmental backgrounds that interact with the variants [6, 7]. Recent GWASs have identified three loci at 18q21 (rs4939827, rs7240004, and rs7229639), which maps to SMAD7, were associated with risk of colorectal cancer [8–10]. SMAD7 is involved in inflammation-related pathways and has been shown to modulate transforming growth factor-β (TGF-β) and Wnt signaling, which are central to the development of carcinogenesis [11–15]. The Smad7 gene encodes an intracellular protein, which interacts with the transforming growth factor (TGF)-β type I receptor, targeting it for degradation in the proteasome, then inhibiting TGF-β1-induced phosphorylation of Smad2/Smad3 [16]. *In vivo* and *in vitro* studies also support the important role of SMAD7 in tumor progression of HCC [17, 18]. Given the role of *SMAD7* in the TGF-β signaling pathway and carcinogenesis of HCC, we performed a case-control study to comprehensively examine 3 loci (rs4939827, rs7240004, and rs7229639), which located at *SMAD7* loci, for their associations with HCC risk and clinicopathologic development in a Han Chinese population, which accounts for 92% of the Chinese population [19, 20].

## RESULTS

The demographic and clinical features of individuals in this population are listed in Table 1. There were no significant differences between cases and controls in terms of the distribution of age, sex, smoking and drinking status. All four SNPs conformed to Hardy–Weinberg proportions in the controls (*p* > 0.05).

**Table 1:** Characteristics of cases and controls in this study

Characteristic	Case (*N* = 1,000)	Control (*N* = 1,000)	*p*-value
**Sex, No.(%)**			
**Male**	620 (62.0%)	612 (61.2%)	0.713
**Female**	380 (38.0%)	388 (38.8%)	
**Age, No**			
**< 50**	523 (52.3%)	521 (52.1%)	0.929
**≥ 50**	477 (47.7%)	479 (47.9%)	
**family history of all cancers**	73 (7.3%)	27 (2.7%)	*P* < 0.01
**Ever smoker**	192 (19.2%)	172 (17.2%)	0.246
**Ever drinker**	199 (19.9%)	201 (20.1%)	0.911
**HBV infection**	221 (22.1%)	84 (8.4%)	*P* < 0.01

*p < 0.05 indicates statistical significance.

The genotype distributions and the association between HCC and healthy controls with *SMAD7* loci polymorphisms are shown in Table 2. Significant differences between the patients with HCC and the controls were detected for all three SNPs (Table 2). Compared with individuals with the major homogeneous genotype, the adjusted OR for developing HCC ranged from 1.41 (95% CI: 1.06–1.88) to 2.64 (95% CI: 1.30–4.39) among those with the minor homogeneous genotype or heterogeneous genotype. Each copy of minor allele was associated with a 1.24–1.36 fold increased risk of HCC. We also conducted sensitivity analyses to exclude the subject with family history of all cancers, as well as add smoking and drinking status to the adjustment variables, however, the results didn't changed materially. Stratified analyses by HBV status, smoking and drinking status were presented in Table 3. All the significant trend kept during the subjects of HBV negative, non-smokers and non-drinkers. However, due to the insufficient statistical power, the trend didn't keep in HBV positive subjects, smokers and drinkers.

**Table 2:** Association between SMAD7 SNPs and HCC risk

	Cases (*N* = 1,000)	Controls (*N* = 1,000)	adjusted OR*
rs4939827			
CC	555	610	1.00 (reference)
CT	325	297	1.20 (0.99–1.46)
TT	120	93	1.42 (1.06–1.90)
T vs C			1.24 (1.07–1.42)
P trend			**3.2 × 10^−3^**
rs7240004			
GG	775	820	1.00 (reference)
AG	200	170	1.24 (0.99–1.56)
AA	25	10	2.64 (1.30–4.39)
A vs G			1.36 (1.12–1.66)
P trend			**2.4 × 10^−3^**
rs7229639			
GG	643	700	1.00 (reference)
AG	235	206	1.24 (1.00–1.54)
AA	122	94	1.41 (1.06–1.88)
A vs G			1.28 (1.10–1.49)
P trend			**1.1 × 10^−3^**

* Adjusted for age, gender, family history of cancer and HBV infection status.

**Table 3:** Stratified analyses of association between SMAD7 SNPs and HCC risk

Variables	rs4939827 (T vs C)	rs7240004 (A vs G)	rs7229639 (A vs G)
**HBV infection**			
Positive	1.23 (0.82–1.86)	1.36 (0.75–2.44)	1.28 (0.82–1.99)
P trend	0.309	0.303	0.264
Negative	1.24 (1.06–1.44)	1.36 (1.09–1.69)	1.28 (1.09–1.51)
P trend	**6.7 × 10^−3^**	**5.2 × 10^−3^**	**2.8 × 10^−3^**
**Smoking status**			
Smokers	1.24 (0.89–1.72)	1.36 (0.85–2.17)	1.28 (0.90–1.83)
P trend	0.210	0.198	0.167
Non-smokers	1.24 (1.06–1.46)	1.36 (1.09–1.70)	1.28 (1.09–1.52)
P trend	**7.6 × 10^−3^**	**6.1 × 10^−3^**	**3.2 × 10^−3^**
**Drinking status**			
Drinkers	1.24 (0.90–1.70)	1.36 (0.87–2.12)	1.28 (0.88–1.90)
P trend	0.174	0.175	0.181
Non-drinkers	1.24 (1.06–1.45)	1.36 (1.09–1.70)	1.28 (1.09–1.52)
P trend	**7.7 × 10^−3^**	**6.7 × 10^−3^**	**3.3 × 10^−3^**

As shown in Table 4, we also analyzed the role of *SMAD7* loci polymorphisms in the clinical TNM stage, primary tumor size, lymph node involvement, distant metastasis, vascular invasion, and Child–Pugh grade. Significant differences were observed between rs4939827 and clinical TNM stage (OR = 1.44, 95% CI: 1.12–1.85, *P* = 4.19 × 10^−3^), vascular invasion (OR = 1.38, 95% CI: 1.03–1.85, *P* = 0.034), as well as rs7240004 and vascular invasion (OR = 1.88, 95% CI: 1.36–2.61, *P* = 1.55 × 10^−4^).

**Table 4:** Age and gender adjusted odds ratio and 95% confidence interval (CI) of HCC clinical status with SMAD7 SNPs

		rs4939827		rs7240004		rs7229639	
		CC/CT+TT	OR (95% CIs)	GG/AG+AA	OR (95% CIs)	GG/AG+AA	OR (95% CIs)
Clinical stage	Stage I/II	300/200	1.00 (reference)	385/115	1.00 (reference)	350/150	1.00 (reference)
	Stage III/IV	255/245	**1.44 (1.12–1.85)**	390/110	0.94 (0.70–1.27)	293/107	0.85 (0.64–1.14)
Tumor size	≤ T2	355/290	1.00 (reference)	492/153	1.00 (reference)	402/243	1.00 (reference)
	> T2	200/155	0.95 (0.73–1.23)	283/72	0.82 (0.60–1.12)	241/114	0.77 (0.59–1.01)
Lymph node metastasis	No	530/424	1.00 (reference)	736/218	1.00 (reference)	614/340	1.00 (reference)
	Yes	25/21	1.05 (0.58–1.90)	39/7	0.61 (0.27–1.36)	29/17	1.06 (0.57–1.95)
Distant metastasis	No	527/423	1.00 (reference)	740/210	1.00 (reference)	613/337	1.00 (reference)
	Yes	28/22	0.97 (0.55–1.74)	35/15	1.51 (0.81–2.81)	30/20	1.21 (0.68–2.17)
Vascular invasion	No	443/330	1.00 (reference)	620/153	1.00 (reference)	506/267	1.00 (reference)
	Yes	112/115	**1.38 (1.03–1.85)**	155/72	**1.88 (1.36–2.61)**	137/90	1.24 (0.92–1.69)
Child–Pugh grade	A	435/333	1.00 (reference)	600/168	1.00 (reference)	495/283	1.00 (reference)
	B or C	120/112	1.22 (0.91–1.64)	175/57	1.16 (0.82–1.64)	148/82	0.97 (0.71–1.32)

To explore the cumulative effect of the three susceptibility SNPs, we established a GRS by summing the risk alleles (Table 5). The GRS was significantly associated with increased risk of HCC and vascular invasion. Compared with subjects with GRs ≤ 3, those with GRS > 3 have an 1.56 fold increased risk of HCC (95% CI: 1.30–1.86, *P* = 7.84 × 10^−7^), as well as 1.63 fold increased risk of vascular invasion (95% CI: 1.21–2.20, *P* = 1.35 × 10^−3^).

**Table 5:** Association of genetic risk score with HCC risk and its clinical status

		GRS	
		≤ 3/> 3	OR (95% CIs)
HCC risk	Controls	600/400	1.00 (reference)
	Cases	490/510	**1.56 (1.30–1.86)**
Clinical stage	Stage I/II	242/258	1.00 (reference)
	Stage III/IV	248/252	0.95 (0.74–1.22)
Tumor size	≤ T2	315/330	1.00 (reference)
	> T2	175/180	0.98 (0.76–1.27)
Lymph node metastasis	No	369/385	1.00 (reference)
	Yes	21/25	1.14 (0.63–2.07)
Distant metastasis	No	365/385	1.00 (reference)
	Yes	25/25	0.94 (0.53–1.68)
Vascular invasion	No	400/373	1.00 (reference)
	Yes	90/137	**1.63 (1.21–2.20)**
Child–Pugh grade	A	377/391	1.00 (reference)
	B or C	113/119	1.02 (0.75–1.36)

## DISCUSSION

It is known that the contribution of risk alleles to HCC risk may vary between populations. This phenomenon may be due to differences in allelic frequencies or specific linkage disequilibrium (LD) structures, or because of additional genetic factors or environmental backgrounds may influence the effect of these genetic variants [25, 26]. In current study, we observed statistically significant associations for the three *SMAD7* loci (rs4939827, rs7240004, and rs7229639) and HCC risk. We also found that significant differences were observed between rs4939827 and clinical TNM stage and vascular invasion, as well as rs7240004 and vascular invasion. To our knowledge, this should be the first to investigate the relationship between HCC risk and *SMAD7* locipolymorphisms.

TGF-β pathway regulates growth inhibition and apoptosis and plays an important role in cancer initiation and progressions [27, 28]. This study highlights the potential importance of the TGF-β genetic polymorphisms was associated with HCC carcinogenesis. These data provide further evidence that common genetic variants in *SMAD7* may confer susceptibility to HCC, particularly in the Chinese Han population. More research is warranted to confirm these findings and functionally characterize the *SMAD7* variants. Among the three *SMAD7* loci studied, both rs4939827 and rs7229639 were located in the intron region of the *SMAD7* gene, while rs7240004 were located in the 3′ downstream of the *SMAD7* gene. Using HaploReg V4.1 [29, 30], we found that about 16 Motifs changed for the three variants, especially for rs4939827, resulted in 11 altered motifs. While using RNA structure website (http://rna.urmc.rochester.edu/RNAstructureWeb/), we found the variation these loci with resulted in the change of secondary structures and influence on the stabilities of *SMAD7* RNA, which will then influence the functions of *SMAD7.* Vascular invasion is the most important predictor of survival in HCC, thus, a link to vascular invasion means these 2 SNPs potentially could be the predictor of survival in HCC [31].

*In vivo* and *in vitro* studies also support the important role of *SMAD7* in tumor progression of HCC. Feng et al. found YB-1/Smad7 could interfere with anti-proliferative/tumor-suppressive TGF-β actions in a subgroup of HCC cells, which may facilitate aspects of tumor progression [17]. High miR-520g expression promotes HCC cell mobility and EMT by targeting *SMAD7*, which is correlated with reduced survival in HCC patients [32]. Loss of Smad7 can enhance susceptibility to HCC, and *SMAD7* suppresses HCC cell growth by inhibiting proliferation and G1-S phase transition and inducing apoptosis through attenuation of NFKB and TGFβ signaling [33].

This study had several limitations. First, selection bias, which is an intrinsic defect of case-control study, might have occurred when the sampling is not random within the subpopulations of cancer and cancer-free subjects; Second, in spite of the relatively large sample size, the power to elucidate gene–environment interactions was limited because of the small magnitudes of the overall associations. In conclusion, polymorphisms in the *SMAD7* were loci highly associated with HCC risk among Chinese population. The combined effects of *SMAD7* loci polymorphisms with environmental carcinogens significantly increase the risk of developing HCC, as well as clinicopathologic development.

## MATERIALS AND METHODS

### Study population

This study was conducted as a population-based case-control study among Chinese Han population. The case population was comprised of newly diagnosed HCC patients which were recruited from affiliated Lishui Hospital of Zhejiang University and the first affiliated hospital of Chongqing Medical University, while control subjects were randomly recruited from the health centers during the same period. The control population was matched with the case population based upon age and gender. All cases had histologically confirmed HCC. For each participant, a standard questionnaire was used to collect demographic information, including age, sex, HBV infection status, smoking status, alcohol use, and family history of all cancer. All subjects signed informed consent forms. Blood (5 ml) was collected from each subject according to the study protocol approved by the Clinical Research Ethics Committee.

### Genotyping

Genomic DNA was extracted from the peripheral blood using the GoldMag Whole Blood Genomic DNA Extraction kit according to the manufacturer's instructions. DNA concentrations were measured using a NanoDrop 2000 (Thermo Scientific, Waltham, MA, USA). A Sequenom Mass ARRAY mass spectrometry analyzer (Sequenom, San Diego, CA, USA) was used for genotyping, and data were managed using Sequenom Typer 4.0 Software (Sequenom, San Diego, CA, USA) [21, 22].

### Statistical analyses

Differences in the distribution of selected demographic variables between HCC cases and cancer-free controls were evaluated using the Student's *t*-test for continuous variables or Pearson's *χ*^2^ test for categorical variables. The association for each of the *SMAD7* loci genotypes and haplotypes was evaluated using unconditional logistic regression models. In controls, each SNP was tested to determine whether it fit with the Hardy–Weinberg equilibrium (HWE). Odds ratios (OR) and 95% confidence intervals (CI) were calculated using unconditional logistic regression analyses adjusted for age, gender, family history of all cancer and HBV infection status [23], and the most common control homozygote was used as reference. LD of the candidate SNPs was analyzed using Haploview v4.2 [24]. To measure the cumulative effect of multiple genetic risk variants, we calculated a genetic risk score (GRS) by summing the number of risk alleles at each locus (0, 1, or 2). All *p*-values reported in this study were two-tailed and *p*-values less than 0.05 were considered statistically significant.
